# Epigenetic modulation in chronic hepatitis B virus infection

**DOI:** 10.1007/s00281-020-00780-6

**Published:** 2020-03-17

**Authors:** Maura Dandri

**Affiliations:** 1 I. Department of Internal Medicine, Center for Internal Medicine, University Medical Center Hamburg-Eppendorf, Martinistr. 52, 20246 Hamburg, Germany; 2 German Center for Infection Research (DZIF), Hamburg-Lübeck-Borstel-Riems Site, Hamburg, Germany

**Keywords:** HBV, cccDNA, Minichromosome, HBx, Interferon

## Abstract

The human hepatitis B virus (HBV) is a small-enveloped DNA virus causing acute and chronic hepatitis. Despite the existence of an effective prophylactic vaccine and the strong capacity of approved antiviral drugs to suppress viral replication, chronic HBV infection (CHB) continues to be a major health burden worldwide. Both the inability of the immune system to resolve CHB and the unique replication strategy employed by HBV, which forms a stable viral covalently closed circular DNA (cccDNA) minichromosome in the hepatocyte nucleus, enable infection persistence. Knowledge of the complex network of interactions that HBV engages with its host is still limited but accumulating evidence indicates that epigenetic modifications occurring both on the cccDNA and on the host genome in the course of infection are essential to modulate viral activity and likely contribute to pathogenesis and cancer development. Thus, a deeper understanding of epigenetic regulatory processes may open new venues to control and eventually cure CHB. This review summarizes major findings in HBV epigenetic research, focusing on the epigenetic mechanisms regulating cccDNA activity and the modifications determined in infected host cells and tumor liver tissues.

## Introduction

Liver disease associated with persistent infection with the hepatitis B virus (HBV) continues to be a major health problem of global impact with at least 240 million people chronically infected worldwide. Even if HBV is not directly cytopathic for the infected cell, the outcome of HBV infection appears to be determined by a complex network of viral and host factors interacting at different levels. The infection can lead to a wide spectrum of liver disease, spanning from acute resolving infection to chronic hepatitis B (CHB) with different grades of hepatitis, which often progresses to liver cirrhosis and hepatocellular carcinoma (HCC) [1]. In spite of the existence of an effective prophylactic vaccine and of approved antiviral regimens, such as the use of nucleos(t)ide analogues (NAs), which efficiently suppress viral replication, resolution of infection is rarely achieved [2]. Both the stability of the persistent viral form, the covalently closed circular HBV DNA (cccDNA), in the hepatocyte nucleus and the inability of the immune system to resolve CHB represent key mechanisms of HBV persistence. NAs potently block the viral reverse transcriptase and long-term treatments have shown to prevent disease progression in most patients and to reduce the risk of HCC in non-cirrhotic patients. However, discontinuation of NAs administration is often bound to the relapse of viral activity, which is mostly due to the inability of these drugs to target directly the viral reservoir cccDNA. Consequently, HBV RNA transcription and production of all viral proteins persist. In contrast, interferon alpha bears both immune modulatory and antiviral effects. In particular, IFN alpha (IFNα) administration was shown to accelerate the degradation of the HBV pregenomic RNA and of core particles in transgenic mice [3, 4] and to induce epigenetic repression of the cccDNA in human hepatocytes both in vitro and in vivo [5]. In its pegylated form, peg-IFNα represents the alternative approved finite treatment for chronic HBV infection, although it is only effective in approximately 20% of patients and its use is limited due to side effects. Thus, there is a strong need to develop therapeutic approaches aiming to achieve a curative strategy that either eliminates or permanently silences the cccDNA [6–8].

The episomal HBV DNA template exists in the nucleus as a minichromosome associated with histones and non-histone proteins. Thus, epigenetic regulation mechanisms, such as DNA methylation, histone modifications, interactions with non-coding RNAs, and chromatin remodeling enzymes, can affect its activity and may have the potential to offer new therapeutic concepts to cure CHB [9]. On the other hand, HBV components may also alter the host genome both genetically and at epigenetic level [10]. Such dysregulation of gene expression is likely to promote liver disease and HCC development.

Despite many years of research in the HBV field, specific aspects of the replication cycle, including the molecular mechanisms regulating cccDNA biology and the interactions that the virus engages with its host, both within the infected human hepatocytes and with the immune system, are still poorly elucidated [11, 12]. Such gap of knowledge is mostly due to the narrow host range and tissue-specificity of HBV, factors that strongly limited the development and availability of robust in vitro and in vivo HBV infection systems.

## HBV replication cycle

**Fig. 1:**
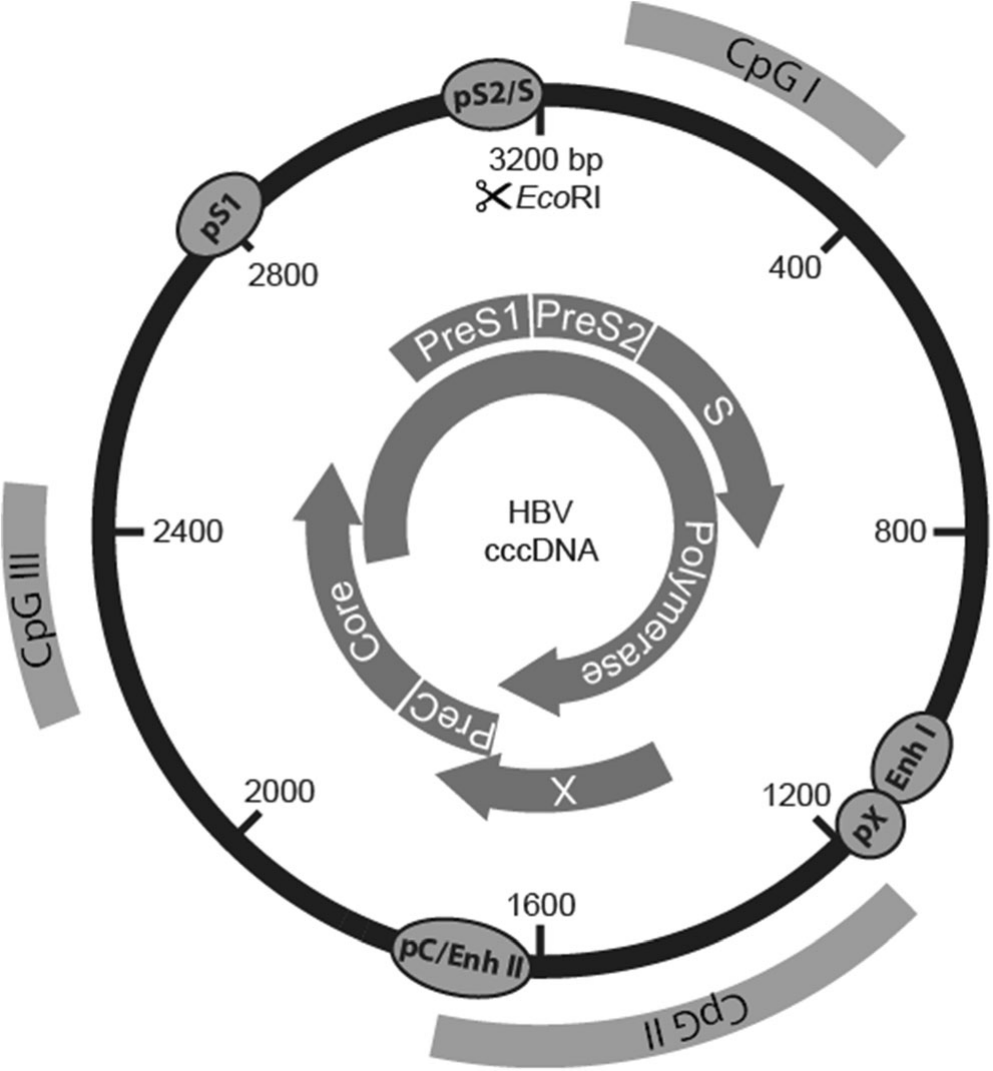
Structure of the HBV genome with the open reading frames (ORFs) shown as curved arrows, the promoters and enhancers, as well as the three predicted CpG islands

Circulating infectious viral particles contain a small circular partially double-stranded DNA (about 3200 nucleotides). Entry of HBV into the human hepatocytes involves its binding to the hepatocyte-specific receptor, the bile acid transporter sodium taurocholate cotransporting polypeptide (NTCP) [13]. Although the steps following viral entry are still poorly characterized, in vitro studies showed that through interactions with nuclear transport receptors and adaptor proteins of the nuclear pore complex, the capsids disintegrate releasing the core capsid subunits and the relaxed circular HBV DNA (rcDNA) genome into the cell nucleus [14]. Even the mechanisms permitting the conversion of the incoming rcDNA to an episomal supercoiled cccDNA molecule that associates with histone and non-histone proteins remain largely unknown. These mechanisms require the DNA repair machinery of the host [15, 16] to remove the covalently attached viral polymerase [17], to complete the positive-strand, and to form a plasmid-like HBV DNA form [11, 18, 19]. The HBV genome is organized in a highly condensed way, where all genes are encoded within largely overlapping open reading frames (ORFs). Of note, all ORFs are identically oriented and are encoded by the negative strand. As shown in Fig. 1, six start codons, four distinct promoters (pS1, pS2, pC, pX), and two transcription-enhancing elements (Enh1 and Enh2) have been identified on the HBV genome. The four major ORFs are (I) preS/S, encoding the three viral surface proteins; (II) precore/core, encoding both the core protein and the non-structural precore protein, from which the secreted e-antigen (HBeAg) is produced; (III) pol, which encodes for the viral polymerase; and (IV) X, coding for the small non-structural regulatory HBx protein.

The circular HBV cccDNA minichromosome utilizes the cellular transcriptional machinery to produce all viral RNAs necessary for protein production and viral replication, which takes place in the cytoplasm after reverse transcription of the over-length (3.5 kb) pre-genomic HBV RNA (pgRNA) [20–22]. Such viral RNA not only serves as templates for the production of new virions but also encodes the viral polymerase and the core proteins that are needed for its encapsidation and reverse transcription. Thus, the pgRNA provides all components required for the production of new HBV DNA-containing nucleocapsids. In contrast, the production of the three envelope proteins (large, middle, and small HBV surface antigen) depends on the transcription of so-called subgenomic HBV RNAs (preS/S) [11]. For the secretion of infectious viral particles, the nucleocapsids are enveloped and secreted from the cell. Even though the molecular mechanisms involved in the release of infectious viral particles are not fully elucidated, HBV egress was shown to occur via multivesicular bodies (MVBs) [23]. Of note, the unique replication mechanism employed by HBV hints that the virus has developed sophisticated strategies both to camouflage its genome as a minichromosome and to produce viral progeny without offering many possibilities to host defense mechanisms to recognize the infection.

Although the tight genomic organization of HBV limits the emergence of variant species, HBV genomic variability is inevitable and attributed to the lack of proofreading by the HBV polymerase, which leads to the emergence of HBV quasi-species with replicative advantages. Such mutations of the HBV DNA genome mostly appear to cluster in particular regions, such as in the preS/S region [24] and in the basal core promoter (BCP) and precore (preC) regions. While all these mutations are associated with the enhanced risk of HCC development, the BCP mutations are strongly associated with high HBV replication levels [25].

Notably, infection studies in ducks and woodchucks revealed that the newly synthesized DNA-containing nucleocapsids are promptly transported into the cell nucleus to build a pool of cccDNA molecules, where up to 50 cccDNA molecules/cell are commonly detected [11]. The intracellular amplification of cccDNA molecules was shown to occur already in the early phases of infection in animals infected with HBV-related viruses. However, lower cccDNA amounts are often measured in liver biopsies of chronically HBV-infected patients [25–27] and in the liver of human chimeric mice (with an average of 1 to 5 copies/cell) [12, 28, 29]. Despite the technical challenges involved in the quantitative determination of HBV cccDNA copies per cell and reports indicating up to 15 copies/cell [30, 31], increasing evidence indicates that different viral and host mechanisms control the pool size of the cccDNA in human hepatocytes [32]. Since the cccDNA minichromosome appears to persist for the entire life span of the infected cells [12], elimination of the cccDNA pool is very challenging. Thus, a strong immune-mediated destruction of the infected hepatocytes [33] or the induction of substantial cccDNA destabilization [34] and/or epigenetic silencing [5] appears necessary to clear HBV infection.

## Chronic HBV infection and liver cancer

In immunocompetent adults, HBV infection generally results in a self-limited, transient liver disease, where viral control is achieved in more than 95% of adults. However, more than 90% of individuals exposed to HBV at birth or in perinatal age become persistently infected [35]. The resolution of an acute self-limited HBV infection requires effective viral recognition and concerted induction of innate and adaptive immune responses with the development of strong and polyclonal CD8+T cell and CD4+T cell responses to HBV proteins [36], whereas in chronic HBV infection, immune responses appear weak and narrowly focused [37]. Of note, HBV does not induce a strong activation of the innate immune system and of interferon-stimulated genes (ISGs) in the early phases of infection [38–40]. Both the unique viral replication process that avoids triggering effective antiviral mechanisms within the hepatocytes and the production of specific viral proteins, like the HBeAg, the regulatory HBx protein, and large amounts of empty subviral particles, appear to contribute to the limited effectiveness of the host antiviral response and persistence of HBV infection [35].

The molecular mechanisms determining long-term pathogenesis and HCC development are not fully elucidated, but they are multi-factorial and many epidemiological and molecular studies have shown that chronic HBV infection represents the main risk factor for HCC development [41]. Current estimates attribute over 50% of HCC cases worldwide to HBV infection [41]. Although most HCCs develop in the context of liver cirrhosis, around one-third of HBV-related HCCs appear to develop in non-cirrhotic livers. Of note, genome-wide analyses and next generation sequencing have indicated that dysregulation of signaling pathways, expression of microRNAs [42], epigenetic changes, and chromatin remodeling occur early in the natural history of HBV-associated tumor development [43].

HBV-driven HCC appears to develop through indirect and direct mechanisms that over the years predispose cell transformation. Such mechanisms include (1) cellular stress and turnover induced by chronic inflammatory processes attempting to clear the infection; (2) modifications of the host genome due to the integration of HBV DNA sequences and to epigenetic alterations; (3) prolonged expression of viral proteins with oncogenic potential, such as the regulatory HBx protein, and the accumulation of altered versions of the HBV envelope proteins.

Liver inflammation causes cell death and compensatory hepatocyte proliferation. Thus, host immune responses are recognized driving forces of liver cell transformation since cell turnover in the presence of oxidative stress, which typically accompany the inflammatory environment, can favor the accumulation of genetic alterations within the hepatocytes [44]. Moreover, coinfection with the hepatitis C virus (HCV), or with the hepatitis D virus (HDV), which causes the most severe form of chronic viral hepatitis with stronger enhancement of inflammatory cytokines and ISGs in infected cells [40], as well as the exposure to aflatoxin B1, augments the risk of HCC development in CHB [44].

HBV does not need to integrate into the host genome for HBV replication. Nevertheless, integration of HBV DNA sequences occurs frequently and randomly even at early steps of infection and causes significant genetic alterations into cellular chromosomes, such as direct insertional mutagenesis and genomic instability. Since HBV DNA integrations have been associated with changes in genes involved in cell proliferation, differentiation, and survival, such insertions may play an important role in the initiation of hepatocellular carcinogenesis. Moreover, the integration sites can be clonally amplified in the course of hepatocyte turnover and of tumor expansion, while the incidence of viral integrated fragments appears to increase further in the presence of DNA damage [45–48]. Even though cell proliferation is bound to amplify HBV-integrated sequences, hepatocyte division also promotes cccDNA dilution and loss [45]. Additionally, HBV-integrated sequences are frequently rearranged and not compatible with the expression of functional proteins. Moreover, poorly differentiated HCCs often do not express the hepatocyte-specific NTCP receptor [13, 49], thus hindering new infection events. Keeping this in mind, the lack of HBV infection susceptibility and sustained replication in advanced tumor tissues is not entirely surprising. Considering the high incidence of HBV-associated HCCs and that hepatocarcinogenesis relies on multi-step processes, genetic alterations induced by HBV infection are thought to act already in the early phases of tumor development.

HBV has been shown to replicate in a subset of tumor tissues characterized by a weak invasive phenotype and a liver-specific transcriptome signature [50], while truncated forms of the envelope proteins (preS1/S polypeptides) have been reported in HCCs and HCC-derived cell lines and shown to bear enhanced transforming properties [24, 42]. Moreover, HBV-related HCCs often harbor the HBV X gene integrated, even though these integrations appear frequently deleted in the C-terminal portion of the HBx protein [51–54]. Intriguingly, a recent study [55] provided evidence that such HBx deletion variants isolated from HCCs retain their ability to support cccDNA transcription when expressed in vitro in the presence of HBV X-minus mutant genomes. Both HBx wildtype and such variants are believed to participate in cell transformation through the pleiotropic activities of HBx [55].

Although HBx cannot be considered a classical oncogenic viral protein, like the large T antigen of the polyomavirus SV40, over the last two decades, HBx was reported to interfere with several cellular pathways and, in certain experimental settings, to act as a carcinogenic co-factor [56, 57]. DNA transfection experiments involving HBx overexpression showed that this viral protein can act as a mild transactivator of viral elements as well as a wide range of cellular promoters [58]. Studies using HBx mutants have also shown that the transactivation function of HBx resides between aminoacid 52 and 148 [59]. Altogether, there is strong evidence that the non-structural HBx protein can alter key cellular pathways and host proteins involved in transcriptional activation, chromosome organization, DNA repair, and cell proliferation [55, 60], thus augmenting the risk of HCC development.

## Regulation of cccDNA activity

The cccDNA is an episomal DNA with a plasmid-like structure, which is organized as a minichromosome by histone and non-histone proteins [20, 61, 62]. Hence, its function depends on the activity and dynamic interplay of numerous transcription factors, coactivators, corepressors, and chromatin-modifying enzymes [5, 21, 22]. Congruent with the fact that HBV infects primary human hepatocytes, the cccDNA bears binding sites both for ubiquitous and liver-specific transcription factors [63]. Various host factors that are implicated in the activation of hepatic metabolic processes, such as hepatocyte nuclear factors 1, 3, and 4 (HNF1, HNF3, HNF4), the CCAAT-enhancer-binding protein (C/EBP), the retinoid X receptor (RXR), peroxisome proliferator-activated receptors (PPAR), and the farnesoid-X-receptor (FXR), were shown to bind the HBV genome. Their recruitment on the viral minichromosome appears essential for yielding efficient viral gene expression [21, 64]. Also the involvement of transcription factors like TATA-binding protein (TBP), activator protein 1 (AP-1), the cAMP response element binding protein (CREB), and transcriptional coactivators, such as CREB-regulated transcriptional coactivator 1 (CRTC1), has been reported to play a key role in regulating cccDNA transcription (reviewed in [65]). Besides cellular factors, both the viral core (HBc) and HBx proteins are key elements in cccDNA biology and activity. The viral core protein appears to act mainly as a structural component of the cccDNA minichromosome and it is responsible for the reduced nucleosomal spacing on the cccDNA compared with cellular chromatin [20]. Thus, core proteins might also be involved in the regulation of viral transcription.

Independent studies using the woodchuck model [66], cell culture systems, transgenic mouse models [67, 68], and human liver-chimeric mice [69], have unequivocally demonstrated the requirement of HBx to initiate cccDNA-driven transcription of the viral RNAs and to maintain virion productivity. These studies demonstrated that, despite the establishment of the cccDNA minichromosome, HBV RNA transcription was dramatically impaired in cells inoculated with HBV X-minus mutants, thus showing that HBx is essential to promote cccDNA-driven viral transcription. Using a cccDNA-specific chromatin immunoprecipitation (ChIP) assay, Belloni and colleagues could provide first in vitro evidence that HBx is recruited onto the cccDNA minichromosome, while an HBV X-minus mutant appeared impaired in its replication fitness [70]. Consequently, the recruitment of the transcriptional coactivator p300 appeared impaired, cccDNA-bound histones were rapidly hypo-acetylated, and the recruitment of the histone deacetylases HDAC1 and Sirtuin 1 (hSirt1) on the viral minichromosome increased [70]. While different studies point out the importance of the acetylation status of the cccDNA-bound H3/H4 histones in regulating cccDNA activity [71], the recruitment of HBx on the cccDNA needs further confirmation in the setting of natural infection.

**Fig. 2:**
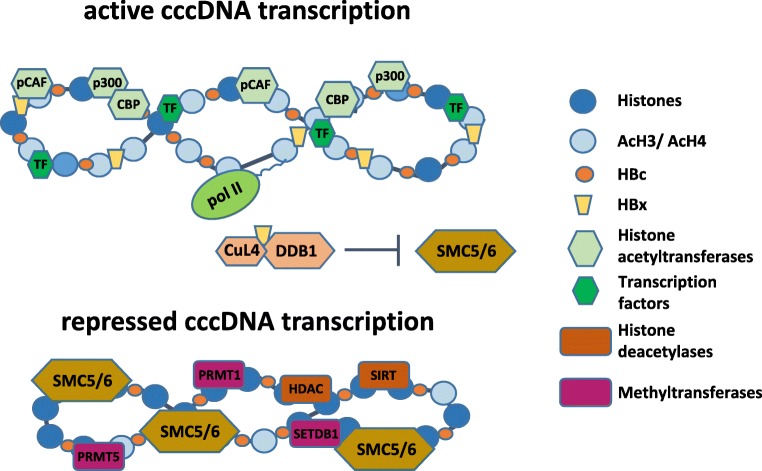
Proposed model of the cccDNA minichromosome with associated host histones with different acetylation status and the viral core protein (HBc). The recruitment of the viral HBx protein, chromatin-modifying enzymes, and transcription factors promotes histone hyperacetylation and active cccDNA transcription. Moreover, HBx promotes cccDNA de-silencing by binding to DDB1 to induce the degradation of the SMC5/6 complex. In the absence of HBx, SMC5/6 is recruited on the cccDNA. A marked hypoacetylation status of the histones accompanies the repressed transcriptional status of the cccDNA

The study of Decorsiere et al. [72] showed that by binding to the damaged DNA binding protein 1 (DDB1), HBx can promote the interaction of the “structural maintenance of chromosomes” (Smc) complex SMC5/6 with a component of the ubiquitin proteasome system, the E3 ubiquitin ligase named Cul4, to trigger ubiquitination and degradation of the SMC5/6 complex (Fig. 2). By binding to the cccDNA, the SMC5/6 complex can act as a host restriction factor suppressing cccDNA transcription. It has been proposed that the initial binding of SMC5/6 complex with cccDNA can block major HBV RNAs (pgRNA, precore mRNA, and surface mRNAs) but not the transcription of HBx mRNA. The early production of HBx could then promote SMC5/6 degradation, thus enabling HBV replication [73]. The SMC5/6 antagonism mediated by HBx seems to be an evolutionarily conserved mechanism found in all mammalian HBV-related viruses since several viruses can exploit the ubiquitin–proteasome system to ensure productive infection. Thus, ubiquitination and degradation of the SMC5/6 complex by the host proteasome machinery, which was demonstrated to occur both in HBV-infected human hepatocytes in vitro and in humanized mice in vivo, represents a new mechanism by which HBx can counteract epigenetic antiviral defenses of the hepatocytes [74]. Of note, integrated HBx variants isolated from some HCC tissues were shown to retain the ability to degrade the SMC5/6 complex [72, 75].

### HBV DNA methylation

DNA methylation often occurs within CpG dinucleotides by the activity of DNA methyltransferases (DNMTs) and is generally associated with transcriptional silencing. The cccDNA contains two to three putative CpG islands, depending on the genotype, which are strategically located in the regulatory elements of the HBV genome (Fig.1). The CpG island 1 overlaps the start site of the S gene; the island 2 encompasses the enhancer I and II, as well as the promoter of the X gene (pX) and of the pregenomic RNA (core promoter, pC); the island 3 covers the region of the pS1 promoter and the start codon of the polymerase gene. While the HBV DNA (rcDNA) in serum and in the cytoplasm of infected cells is mostly unmethylated, the methylation rate of the CpG islands on the nuclear HBV DNA genome may vary and it has been associated with repression of gene transcription in cultures [76, 77]. Moreover, methylation in CpG island 1 appears seldom and to vary among the different HBV genotypes, whereas methylation in CpG islands 2 and 3 appears to be more conserved among genotypes and able to regulate HBV gene expression. Of note, CpG island 2, which overlaps with the promoter of the X gene and the basal core promoter, is minimally methylated in settings where cccDNA transcription is active and replication levels are high. According to this scenario, methylation of CpG islands 2 and 3 has been associated with lower levels of HBV viremia and HBsAg [78]. CpG island 2 methylation was found to be significantly higher also in HBeAg-negative patients, while higher levels of HBV DNA methylation in CpG islands 2 and 3 have been determined in HCC tissues compared with infected and cirrhotic tissues [79–81].

Dissection of the different HBV DNA forms, including rcDNA in the cytoplasm, the nuclear episomal cccDNA, and viral integrated sequences, remains challenging. Nevertheless, integrated HBV DNA was shown to be methylated and silenced in the hepatoma cell line SNU398 [80]. Although viral DNA methylation represents a host defense mechanism aiming at silencing viral gene expression, it is not known whether the mechanisms involved in the methylation of the cccDNA and of integrated sequences differ [9] and may be exploited for therapeutic purposes. Moreover, the study of Jain and colleagues reported the presence of non-CpG cytosine methylation in the HBV DNA obtained from liver tissues [80]. This type of de novo methylation has been reported in pluripotent stem cells and in human papilloma virus (HPV) genomes in cervical cancers [82]. However, non-CpG methylation has been associated with transcription suppression and the significance of these epigenetic marks on the HBV DNA and in liver pathogenesis remains to be explored. Studies in chimeric mice with humanized livers showed genome-wide changes in DNA methylation after infection with either HBV or with HCV in comparison with uninfected animals [83]. Of note, these changes appear to be time-dependent and some of these changes were also observed in HCC patient samples. However, the mice used in the study of Okamoto and colleagues were immune deficient, nonetheless they still retained macrophages and natural killer (NK) cells. Depletion of NK cells appeared to reduce DNA methylation changes, suggesting that some epigenetic events may be triggered by the NK cell activity [83]. Altogether, this study indicates that the chimeric mouse model may serve as an important tool to study virus-induced epigenetic remodeling.

Intriguingly, HBx was described to induce expression of DNA methyltransferases (DNMTs), although the molecular pathways underlying such enhancements remain to be defined [84]. Thus, prolonged expression of HBx is likely to promote epigenetic changes able to influence both the viral cycle and the host cell [60, 85].

### Histone modifications of the cccDNA

Different types of histone-modifying enzymes, such as acetyltransferases (HAT), deacetylases (HDACs), lysine methyltransferases (KMTs), and protein arginine methyltransferases (PRMTs), can alter the histones associated with the cccDNA (Fig. 2). Moreover, DNA methyltransferases (DNMTs) can transfer a methyl group to the cytosine, thus generating a pattern that can be recognized by chromatin modifiers such as HDACs.

Studies in hepatoma cell lines have shown that cccDNA transcription is regulated by the acetylation status of cccDNA-bound histones 3 and 4 (H3 and H4), while data obtained from liver biopsies of HBV-infected patients indicated that histone hypoacetylation and histone deacetylase 1 (HDAC1) recruitment onto the cccDNA correlate with low HBV viremia [71]. In line with this, acetylation of cccDNA-bound H4 was shown to be associated with higher levels of HBV replication, while the use of HDAC inhibitors promoted active acetylation and maintenance of cccDNA activity [71]. Histone acetylation and active cccDNA transcription involve the recruitment of HATs, such as CREB-binding protein (CBP), p300, and the p300/CBP-associated factor (PCAF) on the cccDNA. As mentioned above, HBx protein was shown to play a key role in promoting the recruitment of these HATs to epigenetically regulate cccDNA function [70].

Evidence showing the recruitment of HBx to the cccDNA minichromosome strongly suggests that HBx plays a key role in controlling cccDNA-driven HBV transcription at epigenetic level [21, 70]. Different lines of evidence also indicate that HBx-mediated transcriptional activity relays on the assembly of coactivator and transcription factors complexes involved in transcription and chromatin modulation [86]. In the absence of HBx, cccDNA silencing was associated with the decrease of histone 3 acetylation (H3) and H3K4me3, an increased presence of repressive markers (such as H3K9me2, H3K9me3, H3K27me3), and recruitment of the heterochromatin protein 1 (HP1), a factor that correlates with condensed chromatin [68].

Regarding the organization of the cccDNA minichromosome, Tropberger and colleagues used a chromatin immunoprecipitation-sequencing approach to map post-translational histone modifications across the entire HBV genome. By employing different experimental systems like HBV-infected HepG2-NTCP cells and primary human hepatocytes, as well as human liver biopsies, the authors revealed an unusual chromatin organization of the HBV DNA [22], where the distribution and the levels of active histone modifications appeared comparable with cellular chromatin and were particularly enriched at HBV promotors. However, the study reported an underrepresentation of repressive marks even at silent promoters. For instance, the analysis of HBV-infected cells revealed high levels of activating marks, such as tri-methylation of lysine 4 on H3 (H3K4me3) and a corresponding absence of repressive marks [22].

Using a ChIP-seq approach, Flecken and colleagues recently analyzed fine-needle liver biopsies to define the profile of histone modifications (PTMs) on HBV DNA sequences in patients with different stages of chronic HBV infection [87]. Although the majority of HBV-derived sequences were associated with the activating histone PTM H3K4me3 and such markers correlated with viral transcription levels and HBeAg status, the authors observed strong inter-individual differences in the deposition of histone PTMs in a large proportion of patients. Notably, deposition of classical inhibitory PTM markers (H3K9me3) was detected in around half of the patient biopsy samples, although these could not be linked to reduced levels of viral transcription. Whether such heterogeneity of histone modifications reflects a non-canonical role of PTMs on viral sequences or variations of HBV transcriptional levels occurring at intrahepatic level remains to be investigated.

Given the remarkably different organization of the viral genome, such as its circular conformation, its small size and compact organization of transcripts and regulatory elements, differences in terms of epigenetic regulation are not surprising. However, the precise nature of these differences remains an open question and it appears mandatory to explore whether such unique epigenetic regulation will be amenable to therapeutic intervention.

Intriguingly, experiments performed in vitro and in HBV-infected humanized mice revealed that administration of the therapeutic cytokine IFNα, particularly in its pegylated form (peg-IFNα), can efficiently lower the levels of both pregenomic and subgenomic HBV RNAs. Although the underlying molecular mechanisms leading to the reduction of the major HBV RNAs is not yet elucidated, treatment with peg-IFNα was shown to induce epigenetic modifications of the histones bound to the cccDNA minichromosome [5, 28]. These studies also indicated that IFNα-mediated epigenetic suppression of the cccDNA involved the chromatin remodeling polycomb repressive complex 2 (PRC2) [5, 71]. Moreover, independent chromatin immunoprecipitation-sequencing (ChIP-Seq) experiments demonstrated the reduction of active histone marks upon IFNα administration and such decrease could be even achieved by using a small molecule inhibitor of the responsible histone acetyltransferase, thus proving a functional link between reduced HBV replication and reduced levels of active histone marks [22]. Altogether, these studies provide evidence that IFN-α has the capability to directly contribute to the decline of HBV viremia, amounts of circulating (HBeAg, HBsAg) and intracellular viral antigens (HBcAg) by targeting cccDNA transcription [28].

Particularly interesting are recent in vitro studies showing that treatment with small interfering RNAs (siRNAs) aiming at lowering all HBV RNA levels, including HBx mRNA, leads not only to decreased HBx protein levels but also to the prompt reappearance of the SMC5/6 complex in cultured HBV-infected hepatocytes [88]. Whether and to which extent the siRNA-induced reappearance of the SMC5/6 complex can contribute to cccDNA silencing in vivo is still unknown; however, it is conceivable that also drugs epigenetically suppressing the cccDNA could promote SMC5/6 rebound. Remarkably, preliminary studies have indicated that administration of peg-IFNα in human liver chimeric mice also promotes the reappearance of SMC5/6 complex in human hepatocytes, thus suggesting that IFNα can promote cccDNA silencing by acting at different levels [89].

### Role of non-coding RNAs in cccDNA regulation

MicroRNAs (miRNAs) are short (21–25 nucleotides) sequence-specific non-coding RNA molecules with the function to regulate gene expression. By targeting specific mRNAs, cellular miRNAs can arrest the translation or accelerate RNA degradation of target genes. Interestingly, some miRNAs can also target key epigenetic modifier enzymes. MiRNAs can influence HBV replication directly, by binding to HBV transcripts, or indirectly, by targeting cellular factors involved in HBV replication. In this regard, miR1 was shown to regulate HBV replication by targeting HDAC4 and E2F transcription factor 5, leading to enhanced HBV transcription [90]. Later on, miR449a was shown to target cAMP-responsive element binding protein 5 (CREB5), which in turn induces the expression of FXR, a key factor in bile acid metabolism that can also regulate cccDNA activity in some HBV replication systems [91]. In that study, ectopic expression of miR449a enhanced HBV replication. Of note, miR449a appears to be downregulated in various cancer cells and HCCs. Accordingly, this miRNA appears to act as a tumor suppressor miRNA, which inhibits cell proliferation by targeting key factors involved in cell progression, such as HDAC1 and cyclin D1. Downregulation of such microRNAs may also contribute to the low replication levels of HBV often determined in HBV-derived HCCs.

Yang and colleagues have recently reported that the expression levels of HAT-1, CAF-I, and of the long non-coding RNA (lncRNA) HULC (Highly upregulated in liver cancer) were significantly elevated in infected cell cultures and in the liver of human liver chimeric mice [92]. HAT-1 is mostly involved in the acetylation of newly synthesized histones 3 and 4 and the acetylation pattern generated appears to be recognized by histone chaperons, such as CAF-1 and ASF1, which deposit the histones on synthesized DNA. Thus, this acetyl transferase plays a key role for nucleosome assembly. In line with its function, depletion of HAT-1 mediated by siRNA treatment performed before and at the time of HBV infection reduced the rate of cccDNA establishment in an HBV infection system in vitro [92]. Moreover, the authors showed that HAT-1 is recruited to the cccDNA minichromosome by the lncRNA HULC, which appeared to serve as a scaffold facilitating the assembly of the involved transcriptional regulators. Intriguingly, the HBV core protein appeared to be involved in the HULC-HAT1 interaction, while HBx was shown to enhance the HAT-1 promoter by co-activating the transcription factor Sp1 [92].

## Epigenetic changes on the host

In virus-associated cancers, viral proteins have been shown to participate in epigenetic alterations by disturbing the host DNA methylation system. Localized hypermethylation on promoter areas of tumor suppressor genes (TSGs) can decrease their expression, whereas localized hypomethylation on oncogenes promoters can increase their expression, thus increasing the risk of HCC development [93]. Genomic hypomethylation can also favor chromosome instability, therefore increasing the chance of genome aberration during cell replication. Accumulating evidence indicates that alteration of host DNA methylation occurs early in HBV infection and may contribute to HCC development [94]. Moreover, HBV infection promotes mitochondrial ROS accumulation. Interestingly, such accumulation was shown to induce DNA methylation of genes such as the suppressor of cytokine signaling 3 (SOCS3) [95]. Thus, the methylation of the SOCS3 promoter and subsequent binding of the zinc-finger protein SNAI1 provides a further potential mechanism of epigenetic silencing involved in HBV-mediated carcinogenesis [95]. Of note, the HBx protein was shown to upregulate the expression of DNMT1 and DNMT3A [96] and upregulation of DNMTs was observed in HBV-associated HCC tissues compared with adjacent normal liver tissues. Thus, HBx appears to act as an epigenetic modifying factor in the human liver, which can modulate the transcription of DNA methyltransferases required for normal levels of genomic methylation and maintenance of hypomethylation of TSGs [97]. HBx-promoted hypermethylation of TSGs suggests a novel mechanism by which this promiscuous transactivating protein may accelerate hepatocarcinogenesis.

Genome-wide analysis performed in HBV replicating cell cultures revealed the recruitment of HBx on a large number of target sequences on the host chromatin. These sequences included both protein-coding genes and promoters of non-coding RNAs and siRNAs [98]. Remarkably, pathway analysis indicated an enrichment in genes and siRNAs modulating cell metabolism and chromatin dynamics, as well as genes involved in cellular pathways known to modulate HBV replication, such as calcium transport, SRC, RAS, endocytosis, and the EGF/HGF family [98]. Altogether these studies indicate that HBx is active both on viral and cellular genes, by interacting both with the host chromatin and with numerous host factors in order to generate the most favorable environment for HBV replication and persistence. Moreover, the interaction of HBx with the DNA damage protein 1 (DDB1) and the loss of SMC5/6 represents one of the best-documented HBx-host interactions, which may affect DNA repair, mitosis, and responses to genotoxic stress, thus promoting genomic instability [72, 75]. Intriguingly, SMC5/6 co-localizes with Nuclear Domain 10 (ND10) [99], a dynamic nuclear organelle (nuclear body), implicated in many functions, including regulation of epigenetic events and in innate immune responses. Dysregulation of these nuclear bodies was also observed in HBV-infected cells [60]. Moreover, lower expression of the Nse2 subunit of the SMC5/6 complex has been associated with increased cancer incidence in mice [100]. Thus, it is plausible that HBx-promoted disturbance of the nuclear bodies and degradation of SMC5/6 substantially contribute to the development of HBV-related HCC.

Studies also indicated that the core protein of HBV is not only associated with the cccDNA, but it may also associate with a subset of cellular genes involved in innate immunity, inflammatory responses, and cell proliferation [101]. The role and consequences of such associations in infection in CHB patients await further investigation.

Not only viral proteins such as HBx and HBc but also the episomal viral DNA may interfere with the host chromatin. Moreau and colleagues recently performed chromosome conformation capture experiments (Hi-C) and viral DNA capture in primary human hepatocytes infected either with HBV or adenovirus type 5 to show that these viruses preferentially associate with active chromatin. In particular, they reported a physical contact between HBV DNA and host CpG islands enriched in Cfp1, a factor involved in HBV transcription [10]. Such interactions may facilitate the recruitment of transcription factors needed for viral replication and contribute to the dysregulation of host gene expression.

As mentioned above, the lncRNA HULC was found to be upregulated also in HBV-infected cells [92]. HULC is a 500 nucleotides long transcript found to be upregulated in HCC and studies in hepatoma cell lines indicated its involvement in lipogenesis and angiogenesis, while siRNA knockdown of HULC was shown to deregulate proliferation-related genes [102]. HBV-mediated interference with lncRNAs, such as HULC, represents a further mechanism by which the virus can promote tumor development by deregulating fundamental metabolic and cell cycle regulatory processes that are needed to maintain the hepatocytes highly differentiated.

## Potential for the development of epigenetic therapies

The ability of HBV to build a stable nuclear persistent form, the cccDNA, hampers its eradication. Polymerase inhibitors have clearly shown their ability to lower viremia below threshold of detection in the large majority of treated patients. However, these drugs do not target the cccDNA directly, thus they are inefficient in reducing the cccDNA. Different direct antiviral drugs targeting different steps of HBV infection and/or replication are currently in development or tested in preclinical and clinical trials. These are, for instance, inhibitors of viral entry or release, allosteric modulators of capsid assembly and siRNAs, but their impact on the reduction of already existing cccDNA appears limited [2, 103]. Nevertheless, different strategies have been proposed to lower intrahepatic cccDNA activity in the last few years. By performing cccDNA-ChIP analyses, it could be demonstrated that treatment with IFNα induced hypoacetylation of the cccDNA-bound histones, as well as active recruitment of transcriptional corepressors to the cccDNA [5]. Consequently, cccDNA-driven transcription of the pregenomic and subgenomic viral RNAs was significantly reduced [28]. These results identified a new molecular mechanism whereby therapeutic cytokines, such as pegylated IFNα, besides having immune modulatory effects, can mediate epigenetic repression of the cccDNA transcriptional activity. Moreover, upregulation of cytidine deaminases mediated by high doses of IFNα [104] or the induction of NFκB pathways through antibody-mediated activation of the Lymphotoxin-β-receptor (LTβR) was shown to promote partial cccDNA degradation [34]. Thus, despite the persistence of the cccDNA minichromosome in the hepatocyte nucleus, it is now clear that HBV transcription is regulated through epigenetic modulation involving various host factors and viral proteins. Further understanding of epigenetic mechanisms involved in HBV chronic infection may promote the development of epigenetic therapeutic approaches aiming at inactivating the cccDNA. A realistic goal of CHB therapy is to bring the patients to a clinical situation resembling that of inactive carriers, which have very low or negative HBV DNA and HBsAg levels. Thus, epigenetic suppression of the cccDNA minichromosome would favor the loss of HBsAg and promote achievement of sustained control of HBV infection.

In the field of chronic viral infections, such as HIV and EBV, studies indicate that epigenetic therapies may represent a promising approach [104]. The development of drugs against epigenetic targets and modifying enzymes has gained great attention and some have been approved for the treatment of selected cancers. In principle, epigenetic therapies can focus on targeting enzymes that establish epigenetic marks, the so-called writers, like DNMTs and HATs, or proteins that recognize such marks; the “readers,” which may act as mediators of gene expression by attracting transcription factors; or they may act on the so-called erasers, which remove the epigenetic marks (i.e., HDACs). AGK2, which is an inhibitor of the HDAC SIRT2, was shown to suppress cccDNA transcription in tissue culture under non-cytotoxic conditions [105]. Remarkably, the same study indicated that transcription from integrated HBV DNA sequences was enhanced, thus pointing out a different epigenetic regulation of transcription between episomal and integrated HBV DNA. In a different study, the potential of targeting the histone demethylase KDM5 was explored demonstrating potent reduction of HBV antigens and RNAs associated with histone demethylation (H3K4me3) [106]. The multifunctional protein arginine methyltransferase (PRMT5) appears to induce preferentially H4R3me2 on the cccDNA to repress HBV transcription, thus suggesting that some PTM enzymes may be able to distinguish between the viral episomal template and the host chromatin [107].

Remarkably, epigenetic regulation can also influence the function of immune cells and the release of inflammatory molecules. DNMTs were shown to enhance the expression of major histocompatibility complex class I molecules, which are essential for the presentation of foreign antigens such as HBV epitopes to the T cells [108]. On the other hand, hypo-methylation agents can increase the production of cytokines like interferon gamma and tumor necrosis factor alpha, which can also contribute to cccDNA destabilization [109]. Thus, epigenetic therapy may bear the chance to act both directly on the cccDNA and indirectly by promoting immune functions.

## Conclusion

The ultimate goal of CHB therapy is to eliminate or at least to achieve a complete inactivation of the HBV cccDNA minichromosome. Thus, the development of efficient epigenetic therapies represents a very attractive approach underlining the need to screen compound libraries and available epigenetic drugs for their ability to silence the viral template. While such drugs may potently inactivate the cccDNA, epigenetic alterations of the host genome also bear major risks. In cancer therapy, epigenetic drugs are showing their value. However, many of these drugs are known to have toxic effects and may either lead to the suppression of TSGs or activate latent viral infections. Nevertheless, the different organizations of the episomal HBV DNA in hepatocyte nuclei may offer unique possibilities for the development of drugs specifically targeting viral minichromosome while sparing the host chromatin. Since IFNα treatment induces epigenetic regulation of both ISGs and cccDNA silencing, it is tempting to speculate that by combining interferon with epigenetic drugs, effective synergisms could be achieved. To develop such therapeutic approaches, it will be however important to gain more knowledge on the molecular mechanisms involved in epigenetic regulation of the cccDNA under IFN treatment.

Given the main role of the regulatory HBx protein in initiating and maintaining cccDNA activity, agents able to destabilize or interfere with HBx appear particularly attractive for their potential to silence HBV transcription and to block all different HBx-mediated host alterations that are associated with CHB pathogenesis and liver cancer progression. To achieve such goals, we need to focus research on the comprehension of cccDNA biology and its epigenetic regulation in the setting of infection of the primary human hepatocytes, as well as in the context of liver infection. Accompanied by the new technologies and experimental systems available, we can envision the development of new drugs effectively targeting cccDNA epigenetic regulation in the near future.
